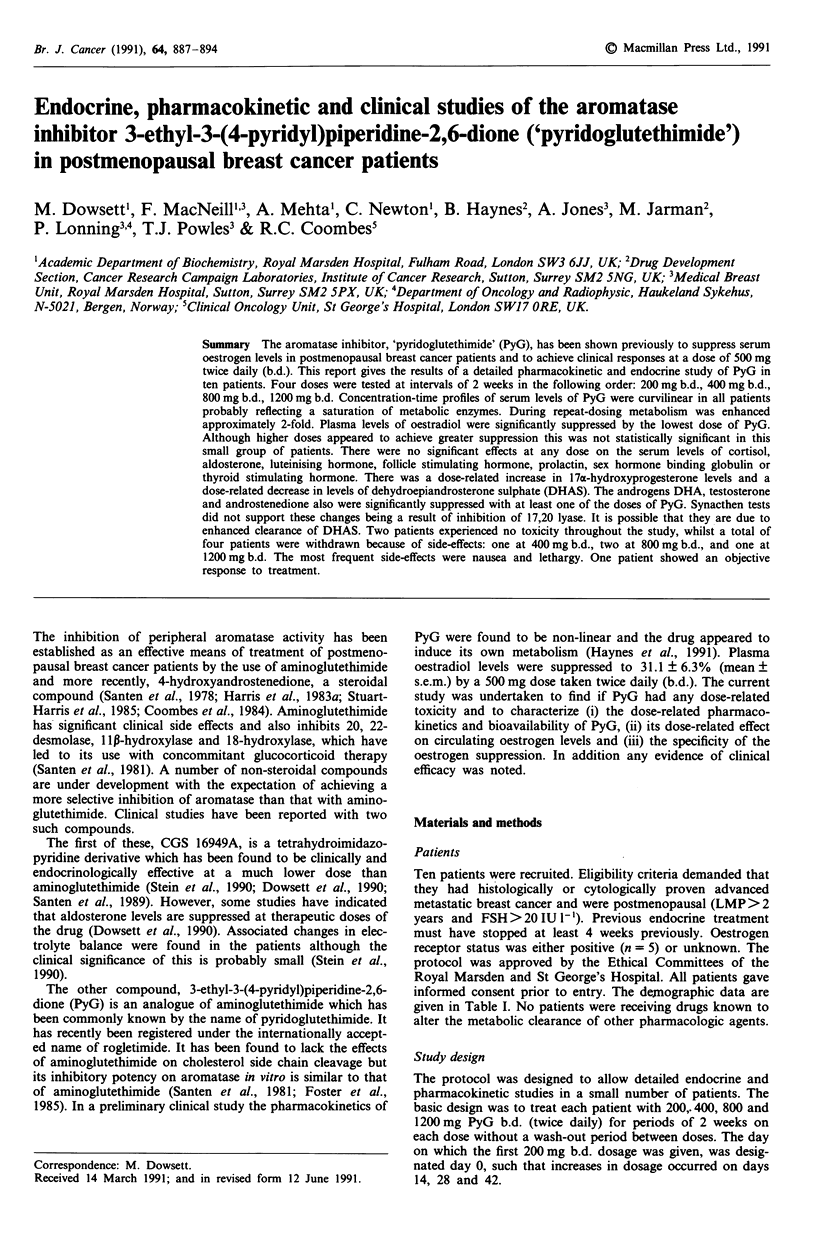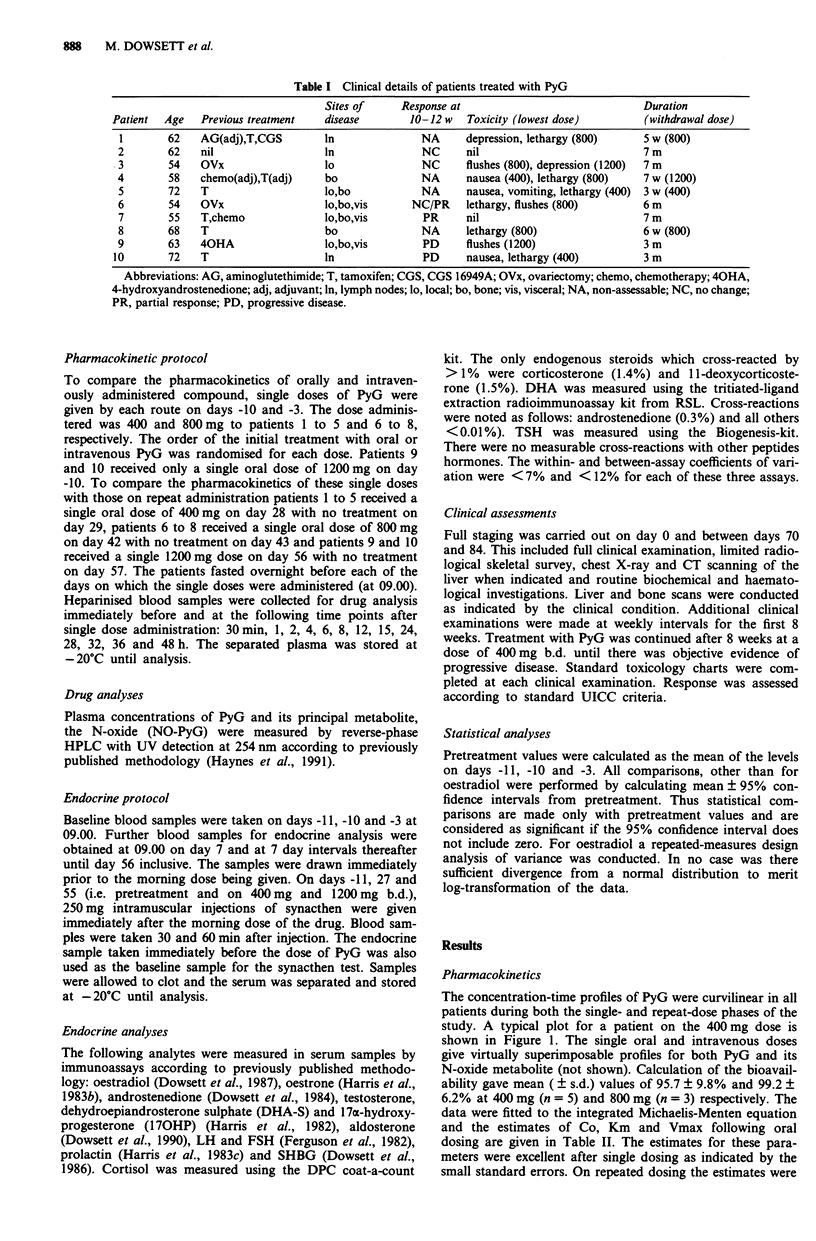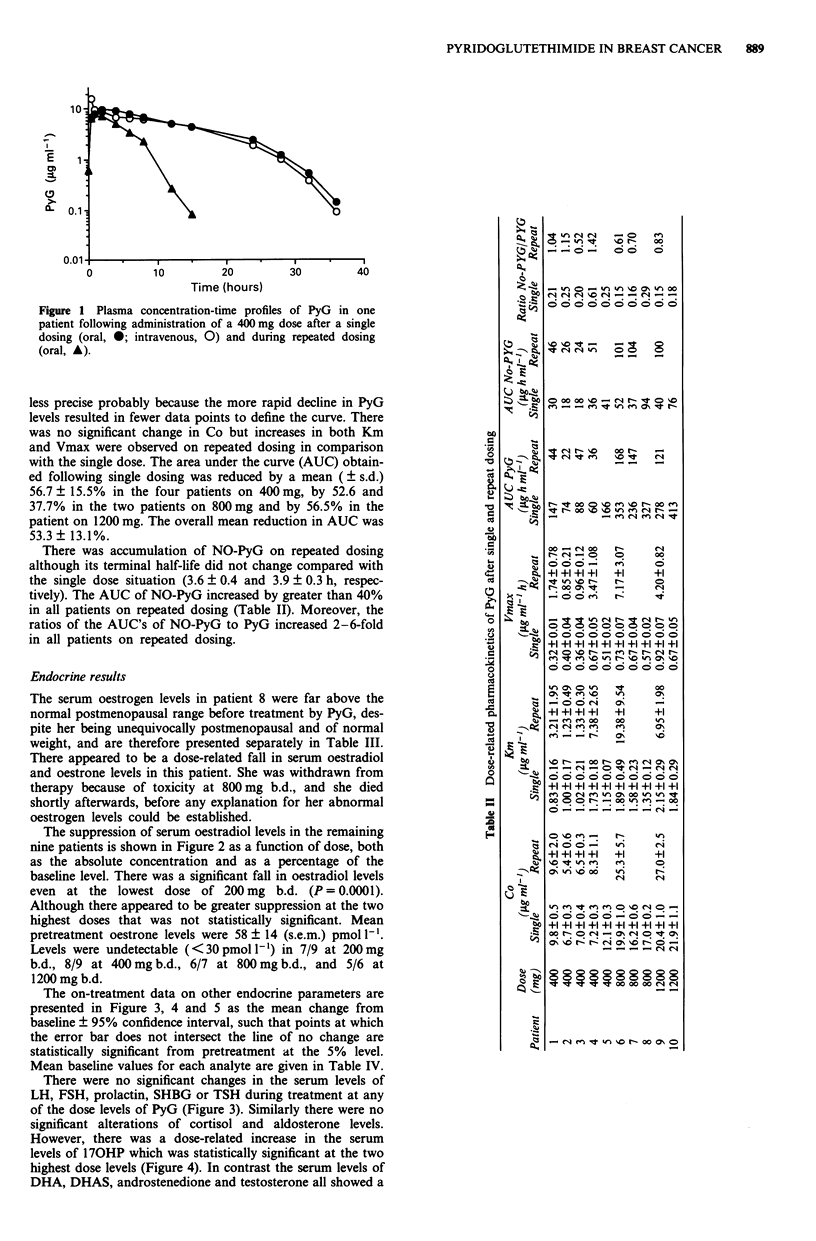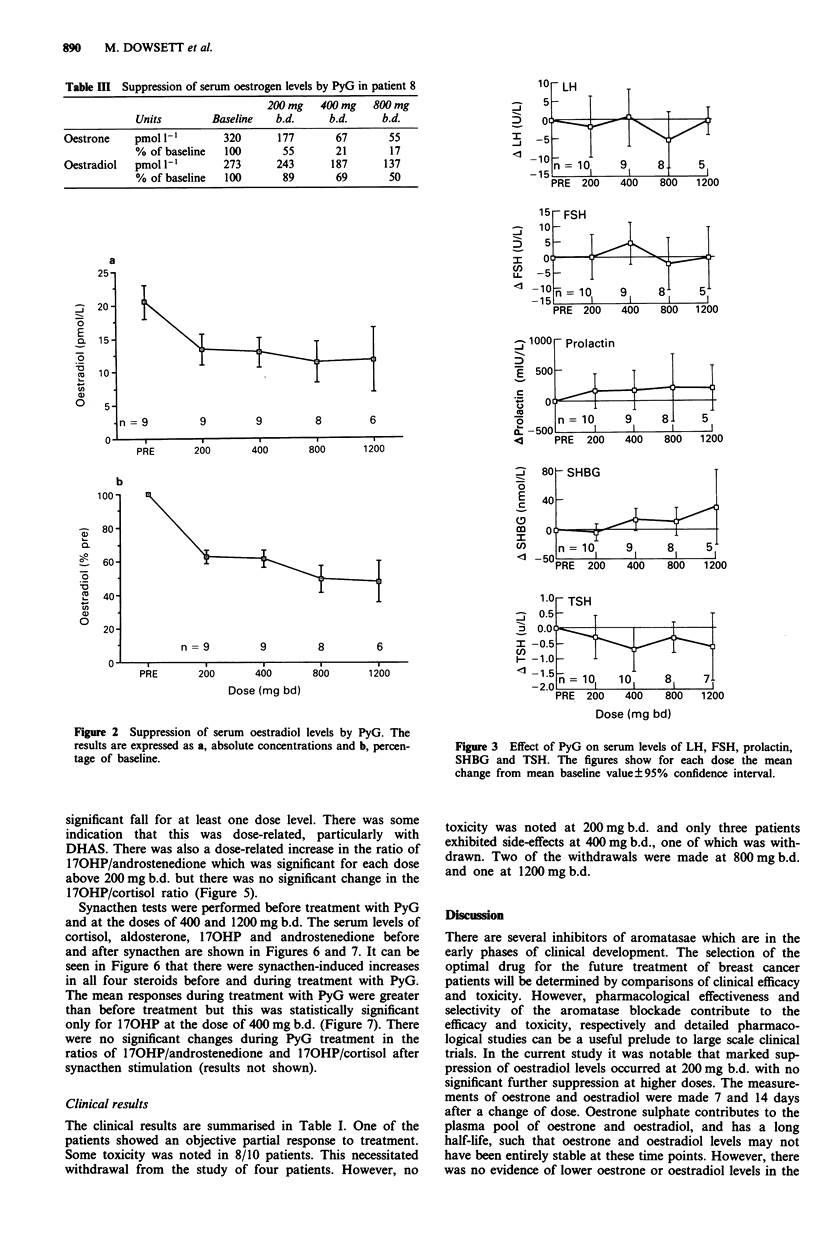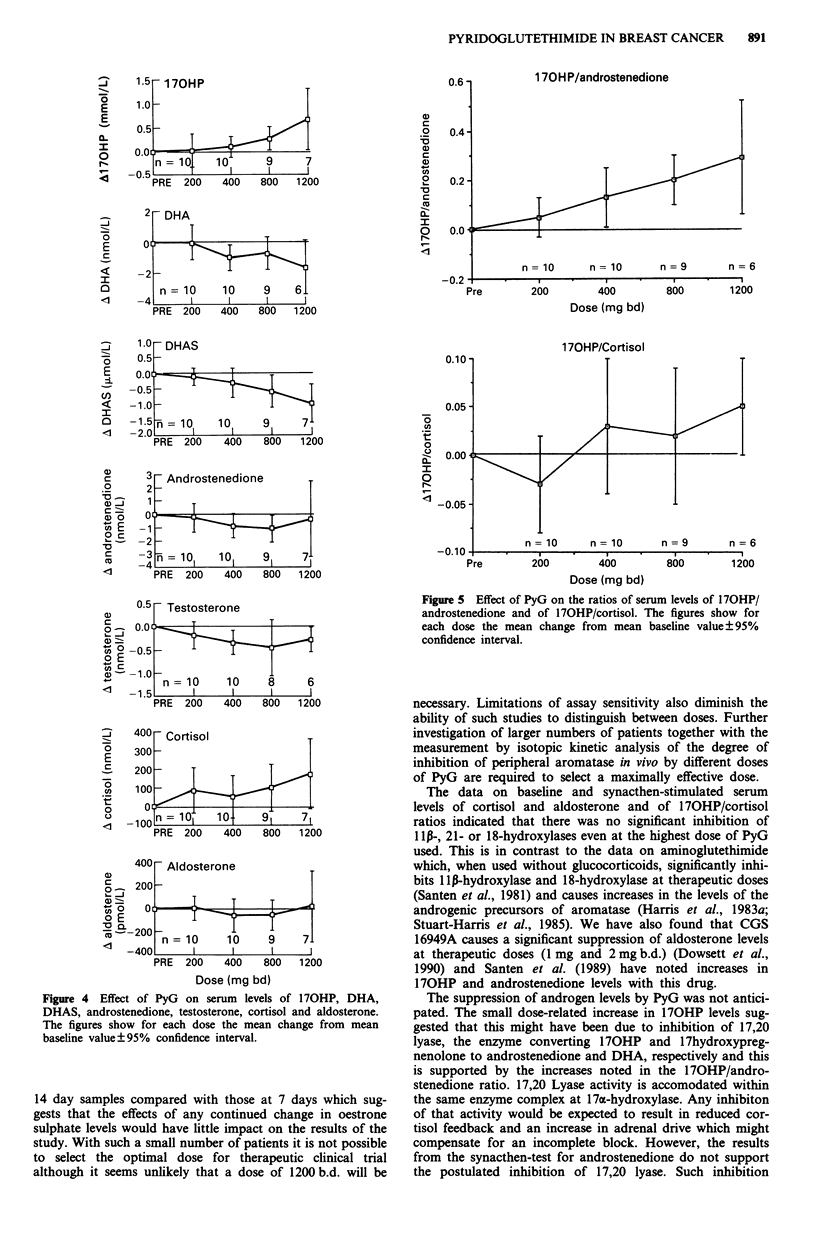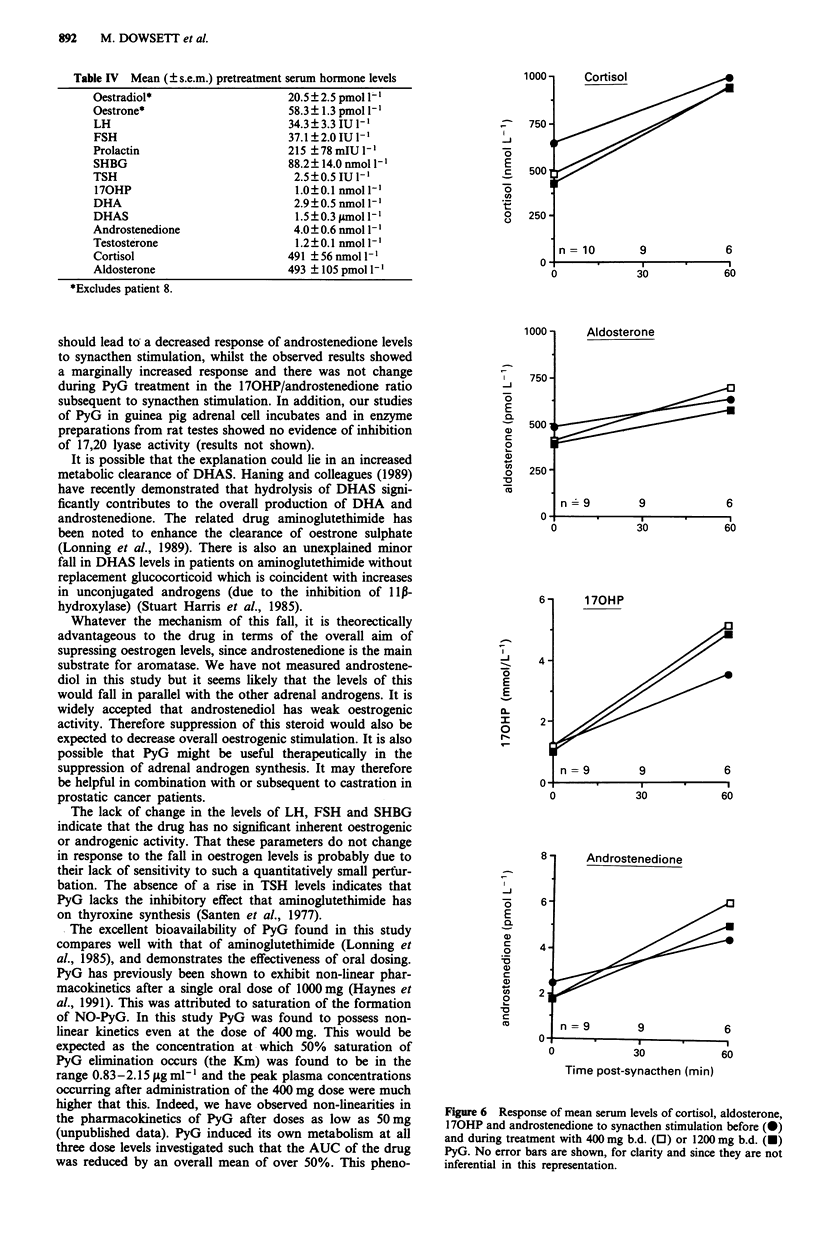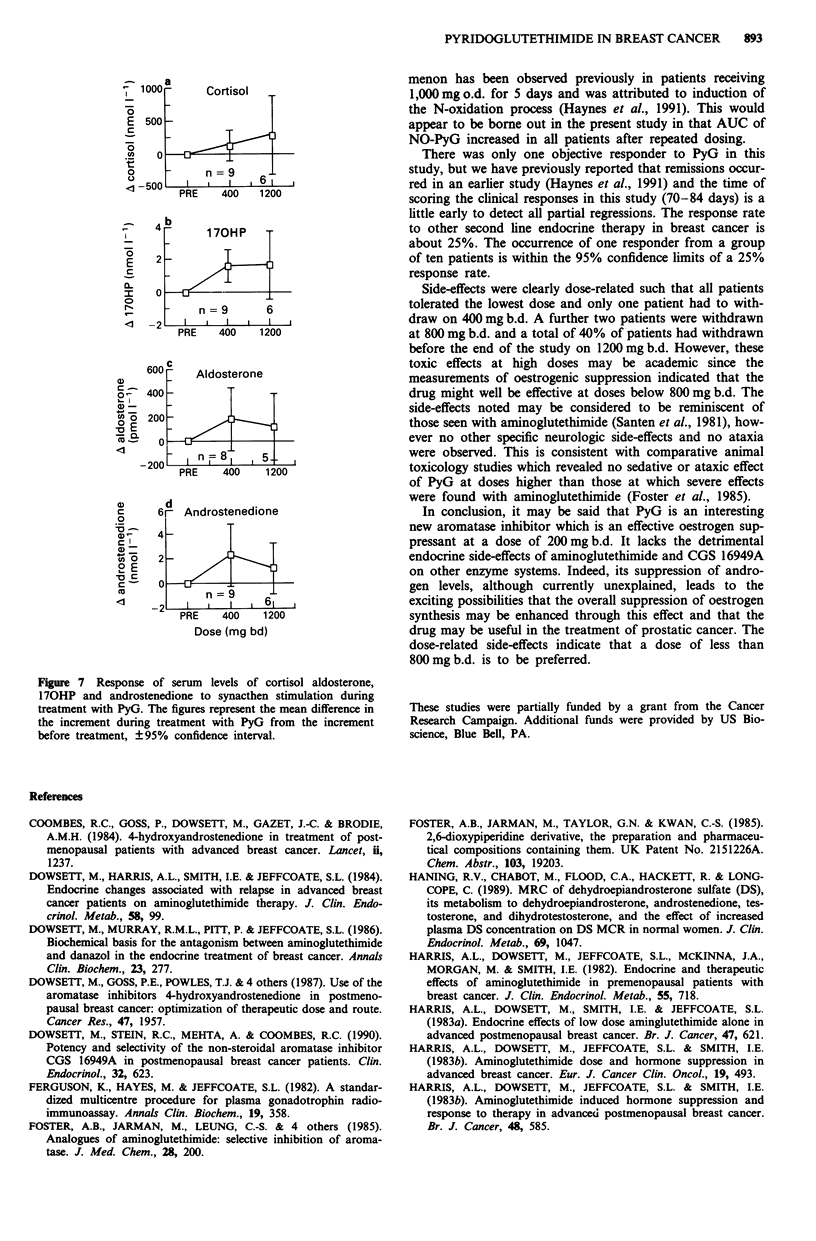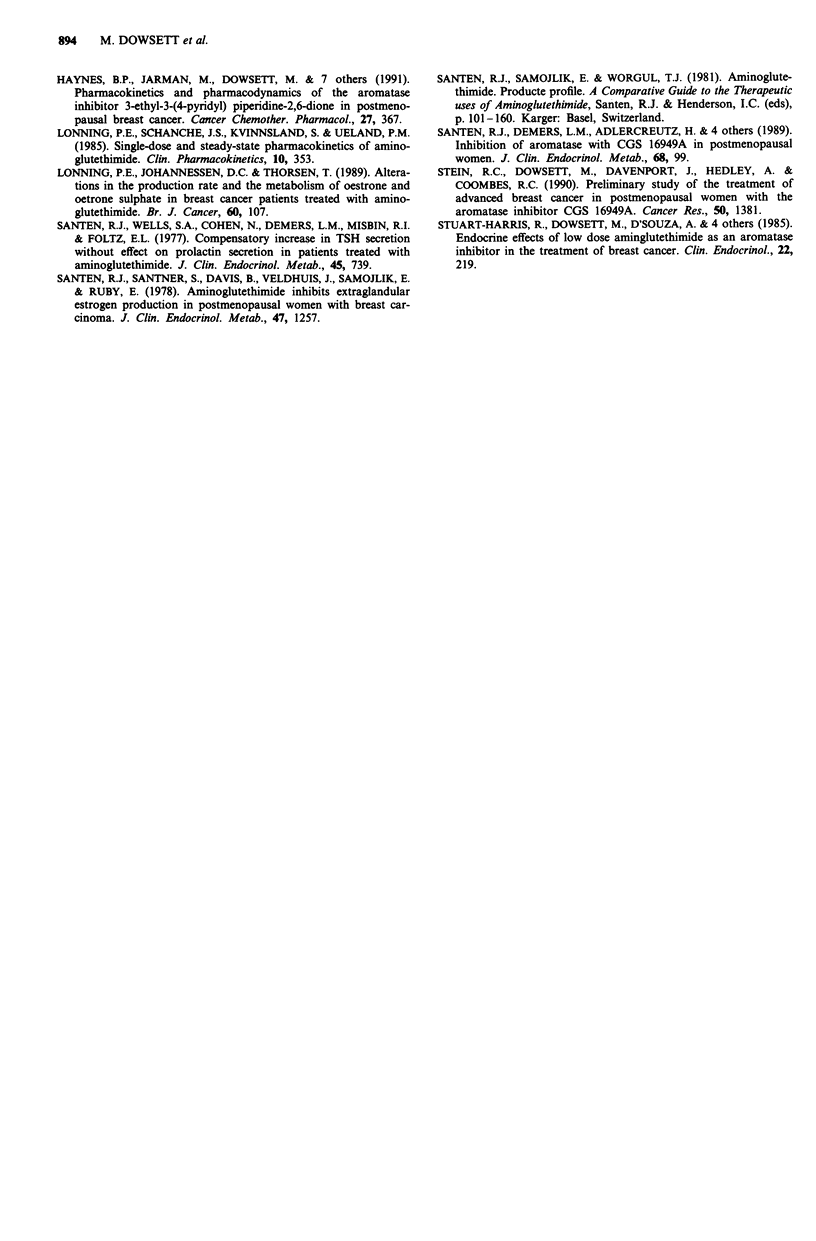# Endocrine, pharmacokinetic and clinical studies of the aromatase inhibitor 3-ethyl-3-(4-pyridyl)piperidine-2,6-dione ('pyridoglutethimide') in postmenopausal breast cancer patients.

**DOI:** 10.1038/bjc.1991.420

**Published:** 1991-11

**Authors:** M. Dowsett, F. MacNeill, A. Mehta, C. Newton, B. Haynes, A. Jones, M. Jarman, P. Lonning, T. J. Powles, R. C. Coombes

**Affiliations:** Academic Department of Biochemistry, Royal Marsden Hospital, London, UK.

## Abstract

The aromatase inhibitor, 'pyridoglutethimide' (PyG), has been shown previously to suppress serum oestrogen levels in postmenopausal breast cancer patients and to achieve clinical responses at a dose of 500 mg twice daily (b.d.). This report gives the results of a detailed pharmacokinetic and endocrine study of PyG in ten patients. Four doses were tested at intervals of 2 weeks in the following order: 200 mg b.d., 400 mg b.d., 800 mg b.d., 1200 mg b.d. Concentration-time profiles of serum levels of PyG were curvilinear in all patients probably reflecting a saturation of metabolic enzymes. During repeat-dosing metabolism was enhanced approximately 2-fold. Plasma levels of oestradiol were significantly suppressed by the lowest dose of PyG. Although higher doses appeared to achieve greater suppression this was not statistically significant in this small group of patients. There were no significant effects at any dose on the serum levels of cortisol, aldosterone, luteinising hormone, follicle stimulating hormone, prolactin, sex hormone binding globulin or thyroid stimulating hormone. There was a dose-related increase in 17 alpha-hydroxyprogesterone levels and a dose-related decrease in levels of dehydroepiandrosterone sulphate (DHAS). The androgens DHA, testosterone and androstenedione also were significantly suppressed with at least one of the doses of PyG. Synacthen tests did not support these changes being a result of inhibition of 17,20 lyase. It is possible that they are due to enhanced clearance of DHAS. Two patients experienced no toxicity throughout the study, whilst a total of four patients were withdrawn because of side-effects: one at 400 mg b.d., two at 800 mg b.d., and one at 1200 mg b.d. The most frequent side-effects were nausea and lethargy. One patient showed an objective response to treatment.


*Br. J. Cancer* (1991), **64**, 887–894 © Macmillan Press Ltd., 1991

### Endocrine, pharmacokinetic and clinical studies of the aromatase inhibitor 3-ethyl-3-(4-pyridyl)piperidine-2,6-dione ('pyridoglutethimide') in postmenopausal breast cancer patients

M. Dowsett[1], F. MacNeill[1,3], A. Mehta[1], C. Newton[1], B. Haynes[2], A. Jones[3], M. Jarman[2], P. Lonning[3,4], T.J. Powles[3] & R.C. Coombes[5]

[1]*Academic Department of Biochemistry, Royal Marsden Hospital, Fulham Road, London SW3 6JJ, UK;* [2]*Drug Development Section, Cancer Research Campaign Laboratories, Institute of Cancer Research, Sutton, Surrey SM2 5NG, UK;* [3]*Medical Breast Unit, Royal Marsden Hospital, Sutton, Surrey SM2 5PX, UK;* [4]*Department of Oncology and Radiophysic, Haukeland Sykehus, N-5021, Bergen, Norway;* [5]*Clinical Oncology Unit, St George's Hospital, London SW17 0RE, UK.*

**Summary** The aromatase inhibitor, 'pyridoglutethimide' (PyG), has been shown previously to suppress serum oestrogen levels in postmenopausal breast cancer patients and to achieve clinical responses at a dose of 500 mg twice daily (b.d.). This report gives the results of a detailed pharmacokinetic and endocrine study of PyG in ten patients. Four doses were tested at intervals of 2 weeks in the following order: 200 mg b.d., 400 mg b.d., 800 mg b.d., 1200 mg b.d. Concentration-time profiles of serum levels of PyG were curvilinear in all patients probably reflecting a saturation of metabolic enzymes. During repeat-dosing metabolism was enhanced approximately 2-fold. Plasma levels of oestradiol were significantly suppressed by the lowest dose of PyG. Although higher doses appeared to achieve greater suppression this was not statistically significant in this small group of patients. There were no significant effects at any dose on the serum levels of cortisol, aldosterone, luteinising hormone, follicle stimulating hormone, prolactin, sex hormone binding globulin or thyroid stimulating hormone. There was a dose-related increase in 17α-hydroxyprogesterone levels and a dose-related decrease in levels of dehydroepiandrosterone sulphate (DHAS). The androgens DHA, testosterone and androstenedione also were significantly suppressed with at least one of the doses of PyG. Synacthen tests did not support these changes being a result of inhibition of 17,20 lyase. It is possible that they are due to enhanced clearance of DHAS. Two patients experienced no toxicity throughout the study, whilst a total of four patients were withdrawn because of side-effects: one at 400 mg b.d., two at 800 mg b.d., and one at 1200 mg b.d. The most frequent side-effects were nausea and lethargy. One patient showed an objective response to treatment.

The inhibition of peripheral aromatase activity has been established as an effective means of treatment of postmenopausal breast cancer patients by the use of aminoglutethimide and more recently, 4-hydroxyandrostenedione, a steroidal compound (Santen *et al.*, 1978; Harris *et al.*, 1983*a*; Stuart-Harris *et al.*, 1985; Coombes *et al.*, 1984). Aminoglutethimide has significant clinical side effects and also inhibits 20, 22-desmolase, 11β-hydroxylase and 18-hydroxylase, which have led to its use with concommitant glucocorticoid therapy (Santen *et al.*, 1981). A number of non-steroidal compounds are under development with the expectation of achieving a more selective inhibition of aromatase than that with aminoglutethimide. Clinical studies have been reported with two such compounds.

The first of these, CGS 16949A, is a tetrahydroimidazopyridine derivative which has been found to be clinically and endocrinologically effective at a much lower dose than aminoglutethimide (Stein *et al.*, 1990; Dowsett *et al.*, 1990; Santen *et al.*, 1989). However, some studies have indicated that aldosterone levels are suppressed at therapeutic doses of the drug (Dowsett *et al.*, 1990). Associated changes in electrolyte balance were found in the patients although the clinical significance of this is probably small (Stein *et al.*, 1990).

The other compound, 3-ethyl-3-(4-pyridyl)piperidine-2,6-dione (PyG) is an analogue of aminoglutethimide which has been commonly known by the name of pyridoglutethimide. It has recently been registered under the internationally accepted name of rogletimide. It has been found to lack the effects of aminoglutethimide on cholesterol side chain cleavage but its inhibitory potency on aromatase *in vitro* is similar to that of aminoglutethimide (Santen *et al.*, 1981; Foster *et al.*, 1985). In a preliminary clinical study the pharmacokinetics of PyG were found to be non-linear and the drug appeared to induce its own metabolism (Haynes *et al.*, 1991). Plasma oestradiol levels were suppressed to 31.1 ± 6.3% (mean ± s.e.m.) by a 500 mg dose taken twice daily (b.d.). The current study was undertaken to find if PyG had any dose-related toxicity and to characterize (i) the dose-related pharmacokinetics and bioavailability of PyG, (ii) its dose-related effect on circulating oestrogen levels and (iii) the specificity of the oestrogen suppression. In addition any evidence of clinical efficacy was noted.

### Materials and methods

### *Patients*

Ten patients were recruited. Eligibility criteria demanded that they had histologically or cytologically proven advanced metastatic breast cancer and were postmenopausal (LMP > 2 years and FSH > 20 IU $l^{-1}$). Previous endocrine treatment must have stopped at least 4 weeks previously. Oestrogen receptor status was either positive ($n = 5$) or unknown. The protocol was approved by the Ethical Committees of the Royal Marsden and St George's Hospital. All patients gave informed consent prior to entry. The demographic data are given in Table I. No patients were receiving drugs known to alter the metabolic clearance of other pharmacologic agents.

### *Study design*

The protocol was designed to allow detailed endocrine and pharmacokinetic studies in a small number of patients. The basic design was to treat each patient with 200, 400, 800 and 1200 mg PyG b.d. (twice daily) for periods of 2 weeks on each dose without a wash-out period between doses. The day on which the first 200 mg b.d. dosage was given, was designated day 0, such that increases in dosage occurred on days 14, 28 and 42.

Correspondence: M. Dowsett.
Received 14 March 1991; and in revised form 12 June 1991.

**Table I** Clinical details of patients treated with PyG

| Patient | Age | Previous treatment | Sites of disease | Response at 10–12 w | Toxicity (lowest dose) | Duration (withdrawal dose) |
|---|---|---|---|---|---|---|
| 1 | 62 | AG(adj),T,CGS | ln | NA | depression, lethargy (800) | 5 w (800) |
| 2 | 62 | nil | ln | NC | nil | 7 m |
| 3 | 54 | OVx | lo | NC | flushes (800), depression (1200) | 7 m |
| 4 | 58 | chemo(adj),T(adj) | bo | NA | nausea (400), lethargy (800) | 7 w (1200) |
| 5 | 72 | T | lo,bo | NA | nausea, vomiting, lethargy (400) | 3 w (400) |
| 6 | 54 | OVx | lo,bo,vis | NC/PR | lethargy, flushes (800) | 6 m |
| 7 | 55 | T,chemo | lo,bo,vis | PR | nil | 7 m |
| 8 | 68 | T | bo | NA | lethargy (800) | 6 w (800) |
| 9 | 63 | 4OHA | lo,bo,vis | PD | flushes (1200) | 3 m |
| 10 | 72 | T | ln | PD | nausea, lethargy (400) | 3 m |

Abbreviations: AG, aminoglutethimide; T, tamoxifen; CGS, CGS 16949A; OVx, ovariectomy; chemo, chemotherapy; 4OHA, 4-hydroxyandrostenedione; adj, adjuvant; ln, lymph nodes; lo, local; bo, bone; vis, visceral; NA, non-assessable; NC, no change; PR, partial response; PD, progressive disease.

*Pharmacokinetic protocol*

To compare the pharmacokinetics of orally and intravenously administered compound, single doses of PyG were given by each route on days -10 and -3. The dose administered was 400 and 800 mg to patients 1 to 5 and 6 to 8, respectively. The order of the initial treatment with oral or intravenous PyG was randomised for each dose. Patients 9 and 10 received only a single oral dose of 1200 mg on day -10. To compare the pharmacokinetics of these single doses with those on repeat administration patients 1 to 5 received a single oral dose of 400 mg on day 28 with no treatment on day 29, patients 6 to 8 received a single oral dose of 800 mg on day 42 with no treatment on day 43 and patients 9 and 10 received a single 1200 mg dose on day 56 with no treatment on day 57. The patients fasted overnight before each of the days on which the single doses were administered (at 09.00). Heparinised blood samples were collected for drug analysis immediately before and at the following time points after single dose administration: 30 min, 1, 2, 4, 6, 8, 12, 15, 24, 28, 32, 36 and 48 h. The separated plasma was stored at −20°C until analysis.

*Drug analyses*

Plasma concentrations of PyG and its principal metabolite, the N-oxide (NO-PyG) were measured by reverse-phase HPLC with UV detection at 254 nm according to previously published methodology (Haynes *et al.*, 1991).

*Endocrine protocol*

Baseline blood samples were taken on days -11, -10 and -3 at 09.00. Further blood samples for endocrine analysis were obtained at 09.00 on day 7 and at 7 day intervals thereafter until day 56 inclusive. The samples were drawn immediately prior to the morning dose being given. On days -11, 27 and 55 (i.e. pretreatment and on 400 mg and 1200 mg b.d.), 250 mg intramuscular injections of synacthen were given immediately after the morning dose of the drug. Blood samples were taken 30 and 60 min after injection. The endocrine sample taken immediately before the dose of PyG was also used as the baseline sample for the synacthen test. Samples were allowed to clot and the serum was separated and stored at −20°C until analysis.

*Endocrine analyses*

The following analytes were measured in serum samples by immunoassays according to previously published methodology: oestradiol (Dowsett *et al.*, 1987), oestrone (Harris *et al.*, 1983*b*), androstenedione (Dowsett *et al.*, 1984), testosterone, dehydroepiandrosterone sulphate (DHA-S) and 17α-hydroxyprogesterone (17OHP) (Harris *et al.*, 1982), aldosterone (Dowsett *et al.*, 1990), LH and FSH (Ferguson *et al.*, 1982), prolactin (Harris *et al.*, 1983*c*) and SHBG (Dowsett *et al.*, 1986). Cortisol was measured using the DPC coat-a-count kit. The only endogenous steroids which cross-reacted by >1% were corticosterone (1.4%) and 11-deoxycorticosterone (1.5%). DHA was measured using the tritiated-ligand extraction radioimmunoassay kit from RSL. Cross-reactions were noted as follows: androstenedione (0.3%) and all others <0.01%). TSH was measured using the Biogenesis-kit. There were no measurable cross-reactions with other peptides hormones. The within- and between-assay coefficients of variation were <7% and <12% for each of these three assays.

*Clinical assessments*

Full staging was carried out on day 0 and between days 70 and 84. This included full clinical examination, limited radiological skeletal survey, chest X-ray and CT scanning of the liver when indicated and routine biochemical and haematological investigations. Liver and bone scans were conducted as indicated by the clinical condition. Additional clinical examinations were made at weekly intervals for the first 8 weeks. Treatment with PyG was continued after 8 weeks at a dose of 400 mg b.d. until there was objective evidence of progressive disease. Standard toxicology charts were completed at each clinical examination. Response was assessed according to standard UICC criteria.

*Statistical analyses*

Pretreatment values were calculated as the mean of the levels on days -11, -10 and -3. All comparisons, other than for oestradiol were performed by calculating mean ± 95% confidence intervals from pretreatment. Thus statistical comparisons are made only with pretreatment values and are considered as significant if the 95% confidence interval does not include zero. For oestradiol a repeated-measures design analysis of variance was conducted. In no case was there sufficient divergence from a normal distribution to merit log-transformation of the data.

### Results

*Pharmacokinetics*

The concentration-time profiles of PyG were curvilinear in all patients during both the single- and repeat-dose phases of the study. A typical plot for a patient on the 400 mg dose is shown in Figure 1. The single oral and intravenous doses give virtually superimposable profiles for both PyG and its N-oxide metabolite (not shown). Calculation of the bioavailability gave mean (± s.d.) values of 95.7 ± 9.8% and 99.2 ± 6.2% at 400 mg ($n = 5$) and 800 mg ($n = 3$) respectively. The data were fitted to the integrated Michaelis-Menten equation and the estimates of Co, Km and Vmax following oral dosing are given in Table II. The estimates for these parameters were excellent after single dosing as indicated by the small standard errors. On repeated dosing the estimates were

**Figure 1** Plasma concentration-time profiles of PyG in one patient following administration of a 400 mg dose after a single dosing (oral, ●; intravenous, ○) and during repeated dosing (oral, ▲).

less precise probably because the more rapid decline in PyG levels resulted in fewer data points to define the curve. There was no significant change in Co but increases in both Km and Vmax were observed on repeated dosing in comparison with the single dose. The area under the curve (AUC) obtained following single dosing was reduced by a mean (± s.d.) 56.7 ± 15.5% in the four patients on 400 mg, by 52.6 and 37.7% in the two patients on 800 mg and by 56.5% in the patient on 1200 mg. The overall mean reduction in AUC was 53.3 ± 13.1%.

There was accumulation of NO-PyG on repeated dosing although its terminal half-life did not change compared with the single dose situation (3.6 ± 0.4 and 3.9 ± 0.3 h, respectively). The AUC of NO-PyG increased by greater than 40% in all patients on repeated dosing (Table II). Moreover, the ratios of the AUC's of NO-PyG to PyG increased 2-6-fold in all patients on repeated dosing.

*Endocrine results*

The serum oestrogen levels in patient 8 were far above the normal postmenopausal range before treatment by PyG, despite her being unequivocally postmenopausal and of normal weight, and are therefore presented separately in Table III. There appeared to be a dose-related fall in serum oestradiol and oestrone levels in this patient. She was withdrawn from therapy because of toxicity at 800 mg b.d., and she died shortly afterwards, before any explanation for her abnormal oestrogen levels could be established.

The suppression of serum oestradiol levels in the remaining nine patients is shown in Figure 2 as a function of dose, both as the absolute concentration and as a percentage of the baseline level. There was a significant fall in oestradiol levels even at the lowest dose of 200 mg b.d. ($P = 0.0001$). Although there appeared to be greater suppression at the two highest doses that was not statistically significant. Mean pretreatment oestrone levels were 58 ± 14 (s.e.m.) pmol $l^{-1}$. Levels were undetectable (<30 pmol $l^{-1}$) in 7/9 at 200 mg b.d., 8/9 at 400 mg b.d., 6/7 at 800 mg b.d., and 5/6 at 1200 mg b.d.

The on-treatment data on other endocrine parameters are presented in Figure 3, 4 and 5 as the mean change from baseline ± 95% confidence interval, such that points at which the error bar does not intersect the line of no change are statistically significant from pretreatment at the 5% level. Mean baseline values for each analyte are given in Table IV.

There were no significant changes in the serum levels of LH, FSH, prolactin, SHBG or TSH during treatment at any of the dose levels of PyG (Figure 3). Similarly there were no significant alterations of cortisol and aldosterone levels. However, there was a dose-related increase in the serum levels of 17OHP which was statistically significant at the two highest dose levels (Figure 4). In contrast the serum levels of DHA, DHAS, androstenedione and testosterone all showed a

**Table II** Dose-related pharmacokinetics of PyG after single and repeat dosing

| Patient | Dose (mg) | Co (µg ml$^{-1}$) Single | Repeat | Km (µg ml$^{-1}$) Single | Repeat | Vmax (µg ml$^{-1}$ h) Single | Repeat | AUC PyG (µg h ml$^{-1}$) Single | Repeat | AUC No-PYG (µg h ml$^{-1}$) Single | Repeat | Ratio No-PYG/PYG Single | Repeat |
|---|---|---|---|---|---|---|---|---|---|---|---|---|---|
| 1 | 400 | 9.8±0.5 | 9.6±2.0 | 0.83±0.16 | 3.21±1.95 | 0.32±0.01 | 1.74±0.78 | 147 | 44 | 30 | 46 | 0.21 | 1.04 |
| 2 | 400 | 6.7±0.3 | 5.4±0.6 | 1.00±0.17 | 1.23±0.49 | 0.40±0.04 | 0.85±0.21 | 74 | 22 | 18 | 26 | 0.25 | 1.15 |
| 3 | 400 | 7.0±0.4 | 6.5±0.3 | 1.02±0.21 | 1.33±0.30 | 0.36±0.04 | 0.96±0.12 | 88 | 47 | 18 | 24 | 0.20 | 0.52 |
| 4 | 400 | 7.2±0.3 | 8.3±1.1 | 1.73±0.18 | 7.38±2.65 | 0.67±0.05 | 3.47±1.08 | 60 | 36 | 36 | 51 | 0.61 | 1.42 |
| 5 | 400 | 12.1±0.3 | | 1.15±0.07 | | 0.51±0.02 | | 166 | | 41 | | 0.25 | |
| 6 | 800 | 19.9±1.0 | 25.3±5.7 | 1.89±0.49 | 19.38±9.54 | 0.73±0.07 | 7.17±3.07 | 353 | 168 | 52 | 101 | 0.15 | 0.61 |
| 7 | 800 | 16.2±0.6 | | 1.58±0.23 | | 0.67±0.04 | | 236 | 147 | 37 | 104 | 0.16 | 0.70 |
| 8 | 800 | 17.0±0.2 | | 1.35±0.12 | | 0.57±0.02 | | 327 | | 94 | | 0.29 | |
| 9 | 1200 | 20.4±1.0 | 27.0±2.5 | 2.15±0.29 | 6.95±1.98 | 0.92±0.07 | 4.20±0.82 | 278 | 121 | 40 | 100 | 0.15 | 0.83 |
| 10 | 1200 | 21.9±1.1 | | 1.84±0.29 | | 0.67±0.05 | | 413 | | 76 | | 0.18 | |

**Table III** Suppression of serum oestrogen levels by PyG in patient 8

| | *Units* | *Baseline* | *200 mg b.d.* | *400 mg b.d.* | *800 mg b.d.* |
|---|---|---|---|---|---|
| Oestrone | pmol $l^{-1}$ | 320 | 177 | 67 | 55 |
| | % of baseline | 100 | 55 | 21 | 17 |
| Oestradiol | pmol $l^{-1}$ | 273 | 243 | 187 | 137 |
| | % of baseline | 100 | 89 | 69 | 50 |

**Figure 2** Suppression of serum oestradiol levels by PyG. The results are expressed as **a**, absolute concentrations and **b**, percentage of baseline.

**Figure 3** Effect of PyG on serum levels of LH, FSH, prolactin, SHBG and TSH. The figures show for each dose the mean change from mean baseline value±95% confidence interval.

significant fall for at least one dose level. There was some indication that this was dose-related, particularly with DHAS. There was also a dose-related increase in the ratio of 17OHP/androstenedione which was significant for each dose above 200 mg b.d. but there was no significant change in the 17OHP/cortisol ratio (Figure 5).

Synacthen tests were performed before treatment with PyG and at the doses of 400 and 1200 mg b.d. The serum levels of cortisol, aldosterone, 17OHP and androstenedione before and after synacthen are shown in Figures 6 and 7. It can be seen in Figure 6 that there were synacthen-induced increases in all four steroids before and during treatment with PyG. The mean responses during treatment with PyG were greater than before treatment but this was statistically significant only for 17OHP at the dose of 400 mg b.d. (Figure 7). There were no significant changes during PyG treatment in the ratios of 17OHP/androstenedione and 17OHP/cortisol after synacthen stimulation (results not shown).

### *Clinical results*

The clinical results are summarised in Table I. One of the patients showed an objective partial response to treatment. Some toxicity was noted in 8/10 patients. This necessitated withdrawal from the study of four patients. However, no toxicity was noted at 200 mg b.d. and only three patients exhibited side-effects at 400 mg b.d., one of which was withdrawn. Two of the withdrawals were made at 800 mg b.d. and one at 1200 mg b.d.

### Discussion

There are several inhibitors of aromatasae which are in the early phases of clinical development. The selection of the optimal drug for the future treatment of breast cancer patients will be determined by comparisons of clinical efficacy and toxicity. However, pharmacological effectiveness and selectivity of the aromatase blockade contribute to the efficacy and toxicity, respectively and detailed pharmacological studies can be a useful prelude to large scale clinical trials. In the current study it was notable that marked suppression of oestradiol levels occurred at 200 mg b.d. with no significant further suppression at higher doses. The measurements of oestrone and oestradiol were made 7 and 14 days after a change of dose. Oestrone sulphate contributes to the plasma pool of oestrone and oestradiol, and has a long half-life, such that oestrone and oestradiol levels may not have been entirely stable at these time points. However, there was no evidence of lower oestrone or oestradiol levels in the

**Figure 4** Effect of PyG on serum levels of 17OHP, DHA, DHAS, androstenedione, testosterone, cortisol and aldosterone. The figures show for each dose the mean change from mean baseline value±95% confidence interval.

14 day samples compared with those at 7 days which suggests that the effects of any continued change in oestrone sulphate levels would have little impact on the results of the study. With such a small number of patients it is not possible to select the optimal dose for therapeutic clinical trial although it seems unlikely that a dose of 1200 b.d. will be

**Figure 5** Effect of PyG on the ratios of serum levels of 17OHP/androstenedione and of 17OHP/cortisol. The figures show for each dose the mean change from mean baseline value±95% confidence interval.

necessary. Limitations of assay sensitivity also diminish the ability of such studies to distinguish between doses. Further investigation of larger numbers of patients together with the measurement by isotopic kinetic analysis of the degree of inhibition of peripheral aromatase *in vivo* by different doses of PyG are required to select a maximally effective dose.

The data on baseline and synacthen-stimulated serum levels of cortisol and aldosterone and of 17OHP/cortisol ratios indicated that there was no significant inhibition of 11β-, 21- or 18-hydroxylases even at the highest dose of PyG used. This is in contrast to the data on aminoglutethimide which, when used without glucocorticoids, significantly inhibits 11β-hydroxylase and 18-hydroxylase at therapeutic doses (Santen *et al.*, 1981) and causes increases in the levels of the androgenic precursors of aromatase (Harris *et al.*, 1983*a*; Stuart-Harris *et al.*, 1985). We have also found that CGS 16949A causes a significant suppression of aldosterone levels at therapeutic doses (1 mg and 2 mg b.d.) (Dowsett *et al.*, 1990) and Santen *et al.* (1989) have noted increases in 17OHP and androstenedione levels with this drug.

The suppression of androgen levels by PyG was not anticipated. The small dose-related increase in 17OHP levels suggested that this might have been due to inhibition of 17,20 lyase, the enzyme converting 17OHP and 17hydroxypregnenolone to androstenedione and DHA, respectively and this is supported by the increases noted in the 17OHP/androstenedione ratio. 17,20 Lyase activity is accomodated within the same enzyme complex at 17α-hydroxylase. Any inhibiton of that activity would be expected to result in reduced cortisol feedback and an increase in adrenal drive which might compensate for an incomplete block. However, the results from the synacthen-test for androstenedione do not support the postulated inhibition of 17,20 lyase. Such inhibition

**Table IV** Mean (±s.e.m.) pretreatment serum hormone levels

| | |
|---|---|
| Oestradiol* | 20.5±2.5 pmol $l^{-1}$ |
| Oestrone* | 58.3±1.3 pmol $l^{-1}$ |
| LH | 34.3±3.3 IU $l^{-1}$ |
| FSH | 37.1±2.0 IU $l^{-1}$ |
| Prolactin | 215 ±78 mIU $l^{-1}$ |
| SHBG | 88.2±14.0 nmol $l^{-1}$ |
| TSH | 2.5±0.5 IU $l^{-1}$ |
| 17OHP | 1.0±0.1 nmol $l^{-1}$ |
| DHA | 2.9±0.5 nmol $l^{-1}$ |
| DHAS | 1.5±0.3 μmol $l^{-1}$ |
| Androstenedione | 4.0±0.6 nmol $l^{-1}$ |
| Testosterone | 1.2±0.1 nmol $l^{-1}$ |
| Cortisol | 491 ±56 nmol $l^{-1}$ |
| Aldosterone | 493 ±105 pmol $l^{-1}$ |

*Excludes patient 8.

should lead to a decreased response of androstenedione levels to synacthen stimulation, whilst the observed results showed a marginally increased response and there was not change during PyG treatment in the 17OHP/androstenedione ratio subsequent to synacthen stimulation. In addition, our studies of PyG in guinea pig adrenal cell incubates and in enzyme preparations from rat testes showed no evidence of inhibition of 17,20 lyase activity (results not shown).

It is possible that the explanation could lie in an increased metabolic clearance of DHAS. Haning and colleagues (1989) have recently demonstrated that hydrolysis of DHAS significantly contributes to the overall production of DHA and androstenedione. The related drug aminoglutethimide has been noted to enhance the clearance of oestrone sulphate (Lonning et al., 1989). There is also an unexplained minor fall in DHAS levels in patients on aminoglutethimide without replacement glucocorticoid which is coincident with increases in unconjugated androgens (due to the inhibition of 11β-hydroxylase) (Stuart Harris et al., 1985).

Whatever the mechanism of this fall, it is theorectically advantageous to the drug in terms of the overall aim of supressing oestrogen levels, since androstenedione is the main substrate for aromatase. We have not measured androstenediol in this study but it seems likely that the levels of this would fall in parallel with the other adrenal androgens. It is widely accepted that androstenediol has weak oestrogenic activity. Therefore suppression of this steroid would also be expected to decrease overall oestrogenic stimulation. It is also possible that PyG might be useful therapeutically in the suppression of adrenal androgen synthesis. It may therefore be helpful in combination with or subsequent to castration in prostatic cancer patients.

The lack of change in the levels of LH, FSH and SHBG indicate that the drug has no significant inherent oestrogenic or androgenic activity. That these parameters do not change in response to the fall in oestrogen levels is probably due to their lack of sensitivity to such a quantitatively small perturbation. The absence of a rise in TSH levels indicates that PyG lacks the inhibitory effect that aminoglutethimide has on thyroxine synthesis (Santen et al., 1977).

The excellent bioavailability of PyG found in this study compares well with that of aminoglutethimide (Lonning et al., 1985), and demonstrates the effectiveness of oral dosing. PyG has previously been shown to exhibit non-linear pharmacokinetics after a single oral dose of 1000 mg (Haynes et al., 1991). This was attributed to saturation of the formation of NO-PyG. In this study PyG was found to possess non-linear kinetics even at the dose of 400 mg. This would be expected as the concentration at which 50% saturation of PyG elimination occurs (the Km) was found to be in the range 0.83–2.15 μg $ml^{-1}$ and the peak plasma concentrations occurring after administration of the 400 mg dose were much higher that this. Indeed, we have observed non-linearities in the pharmacokinetics of PyG after doses as low as 50 mg (unpublished data). PyG induced its own metabolism at all three dose levels investigated such that the AUC of the drug was reduced by an overall mean of over 50%. This pheno-

**Figure 6** Response of mean serum levels of cortisol, aldosterone, 17OHP and androstenedione to synacthen stimulation before (●) and during treatment with 400 mg b.d. (□) or 1200 mg b.d. (■) PyG. No error bars are shown, for clarity and since they are not inferential in this representation.

**Figure 7** Response of serum levels of cortisol aldosterone, 17OHP and androstenedione to synacthen stimulation during treatment with PyG. The figures represent the mean difference in the increment during treatment with PyG from the increment before treatment, ±95% confidence interval.

menon has been observed previously in patients receiving 1,000 mg o.d. for 5 days and was attributed to induction of the N-oxidation process (Haynes *et al.*, 1991). This would appear to be borne out in the present study in that AUC of NO-PyG increased in all patients after repeated dosing.

There was only one objective responder to PyG in this study, but we have previously reported that remissions occurred in an earlier study (Haynes *et al.*, 1991) and the time of scoring the clinical responses in this study (70–84 days) is a little early to detect all partial regressions. The response rate to other second line endocrine therapy in breast cancer is about 25%. The occurrence of one responder from a group of ten patients is within the 95% confidence limits of a 25% response rate.

Side-effects were clearly dose-related such that all patients tolerated the lowest dose and only one patient had to withdraw on 400 mg b.d. A further two patients were withdrawn at 800 mg b.d. and a total of 40% of patients had withdrawn before the end of the study on 1200 mg b.d. However, these toxic effects at high doses may be academic since the measurements of oestrogenic suppression indicated that the drug might well be effective at doses below 800 mg b.d. The side-effects noted may be considered to be reminiscent of those seen with aminoglutethimide (Santen *et al.*, 1981), however no other specific neurologic side-effects and no ataxia were observed. This is consistent with comparative animal toxicology studies which revealed no sedative or ataxic effect of PyG at doses higher than those at which severe effects were found with aminoglutethimide (Foster *et al.*, 1985).

In conclusion, it may be said that PyG is an interesting new aromatase inhibitor which is an effective oestrogen suppressant at a dose of 200 mg b.d. It lacks the detrimental endocrine side-effects of aminoglutethimide and CGS 16949A on other enzyme systems. Indeed, its suppression of androgen levels, although currently unexplained, leads to the exciting possibilities that the overall suppression of oestrogen synthesis may be enhanced through this effect and that the drug may be useful in the treatment of prostatic cancer. The dose-related side-effects indicate that a dose of less than 800 mg b.d. is to be preferred.

These studies were partially funded by a grant from the Cancer Research Campaign. Additional funds were provided by US Bioscience, Blue Bell, PA.

### References

COOMBES, R.C., GOSS, P., DOWSETT, M., GAZET, J.-C. & BRODIE, A.M.H. (1984). 4-hydroxyandrostenedione in treatment of postmenopausal patients with advanced breast cancer. *Lancet*, **ii**, 1237.

DOWSETT, M., HARRIS, A.L., SMITH, I.E. & JEFFCOATE, S.L. (1984). Endocrine changes associated with relapse in advanced breast cancer patients on aminoglutethimide therapy. *J. Clin. Endocrinol. Metab.*, **58**, 99.

DOWSETT, M., MURRAY, R.M.L., PITT, P. & JEFFCOATE, S.L. (1986). Biochemical basis for the antagonism between aminoglutethimide and danazol in the endocrine treatment of breast cancer. *Annals Clin. Biochem.*, **23**, 277.

DOWSETT, M., GOSS, P.E., POWLES, T.J. & 4 others (1987). Use of the aromatase inhibitors 4-hydroxyandrostenedione in postmenopausal breast cancer: optimization of therapeutic dose and route. *Cancer Res.*, **47**, 1957.

DOWSETT, M., STEIN, R.C., MEHTA, A. & COOMBES, R.C. (1990). Potency and selectivity of the non-steroidal aromatase inhibitor CGS 16949A in postmenopausal breast cancer patients. *Clin. Endocrinol.*, **32**, 623.

FERGUSON, K., HAYES, M. & JEFFCOATE, S.L. (1982). A standardized multicentre procedure for plasma gonadotrophin radioimmunoassay. *Annals Clin. Biochem.*, **19**, 358.

FOSTER, A.B., JARMAN, M., LEUNG, C.-S. & 4 others (1985). Analogues of aminoglutethimide: selective inhibition of aromatase. *J. Med. Chem.*, **28**, 200.

FOSTER, A.B., JARMAN, M., TAYLOR, G.N. & KWAN, C.-S. (1985). 2,6-dioxypiperidine derivative, the preparation and pharmaceutical compositions containing them. UK Patent No. 2151226A. *Chem. Abstr.*, **103**, 19203.

HANING, R.V., CHABOT, M., FLOOD, C.A., HACKETT, R. & LONGCOPE, C. (1989). MRC of dehydroepiandrosterone sulfate (DS), its metabolism to dehydroepiandrosterone, androstenedione, testosterone, and dihydrotestosterone, and the effect of increased plasma DS concentration on DS MCR in normal women. *J. Clin. Endocrinol. Metab.*, **69**, 1047.

HARRIS, A.L., DOWSETT, M., JEFFCOATE, S.L., MCKINNA, J.A., MORGAN, M. & SMITH, I.E. (1982). Endocrine and therapeutic effects of aminoglutethimide in premenopausal patients with breast cancer. *J. Clin. Endocrinol. Metab.*, **55**, 718.

HARRIS, A.L., DOWSETT, M., SMITH, I.E. & JEFFCOATE, S.L. (1983*a*). Endocrine effects of low dose aminglutethimide alone in advanced postmenopausal breast cancer. *Br. J. Cancer*, **47**, 621.

HARRIS, A.L., DOWSETT, M., JEFFCOATE, S.L. & SMITH, I.E. (1983*b*). Aminoglutethimide dose and hormone suppression in advanced breast cancer. *Eur. J. Cancer Clin. Oncol.*, **19**, 493.

HARRIS, A.L., DOWSETT, M., JEFFCOATE, S.L. & SMITH, I.E. (1983*b*). Aminoglutethimide induced hormone suppression and response to therapy in advanced postmenopausal breast cancer. *Br. J. Cancer*, **48**, 585.

HAYNES, B.P., JARMAN, M., DOWSETT, M. & 7 others (1991). Pharmacokinetics and pharmacodynamics of the aromatase inhibitor 3-ethyl-3-(4-pyridyl) piperidine-2,6-dione in postmenopausal breast cancer. *Cancer Chemother. Pharmacol.*, **27**, 367.

LONNING, P.E., SCHANCHE, J.S., KVINNSLAND, S. & UELAND, P.M. (1985). Single-dose and steady-state pharmacokinetics of aminoglutethimide. *Clin. Pharmacokinetics*, **10**, 353.

LONNING, P.E., JOHANNESSEN, D.C. & THORSEN, T. (1989). Alterations in the production rate and the metabolism of oestrone and oetrone sulphate in breast cancer patients treated with aminoglutethimide. *Br. J. Cancer*, **60**, 107.

SANTEN, R.J., WELLS, S.A., COHEN, N., DEMERS, L.M., MISBIN, R.I. & FOLTZ, E.L. (1977). Compensatory increase in TSH secretion without effect on prolactin secretion in patients treated with aminoglutethimide. *J. Clin. Endocrinol. Metab.*, **45**, 739.

SANTEN, R.J., SANTNER, S., DAVIS, B., VELDHUIS, J., SAMOJLIK, E. & RUBY, E. (1978). Aminoglutethimide inhibits extraglandular estrogen production in postmenopausal women with breast carcinoma. *J. Clin. Endocrinol. Metab.*, **47**, 1257.

SANTEN, R.J., SAMOJLIK, E. & WORGUL, T.J. (1981). Aminoglutethimide. Producte profile. *A Comparative Guide to the Therapeutic uses of Aminoglutethimide*, Santen, R.J. & Henderson, I.C. (eds), p. 101-160. Karger: Basel, Switzerland.

SANTEN, R.J., DEMERS, L.M., ADLERCREUTZ, H. & 4 others (1989). Inhibition of aromatase with CGS 16949A in postmenopausal women. *J. Clin. Endocrinol. Metab.*, **68**, 99.

STEIN, R.C., DOWSETT, M., DAVENPORT, J., HEDLEY, A. & COOMBES, R.C. (1990). Preliminary study of the treatment of advanced breast cancer in postmenopausal women with the aromatase inhibitor CGS 16949A. *Cancer Res.*, **50**, 1381.

STUART-HARRIS, R., DOWSETT, M., D'SOUZA, A. & 4 others (1985). Endocrine effects of low dose aminglutethimide as an aromatase inhibitor in the treatment of breast cancer. *Clin. Endocrinol.*, **22**, 219.